# Supplementary material for: Improvement of the Bias Stress Stability in 2D MoS2 and WS2 Transistors with a TiO2 Interfacial Layer
Source: Nanomaterials (Basel). 2019 Aug 12;9(8):1155. doi: 10.3390/nano9081155 (PMC6724147; doi:10.3390/nano9081155)
Supplement: Supplementary file 1 [file nanomaterials-09-01155-s001.pdf]

Article

Improvement of the Bias Stress Stability in 2D MoS_2 and WS_2 Transistors with a TiO_2 Interfacial Layer

Woojin Park [1,†], Yusin Pak [2,†], Hye Yeon Jang [1], Jae Hyeon Nam [1], Tae Hyeon Kim [1], Seyoung Oh [1], Sung Mook Choi [3] Yonghun Kim [3] and Byungjin Cho [1,*]

[1] Department of Advanced Material Engineering, Chungbuk National University, Chungdae-ro 1, Seowon-Gu, Cheongju, Chungbuk 28644, Korea
[2] Department of Nanobio Materials and Electronics, GIST, 123 Cheomdan-gwagiro, Buk-gu, Gwangju 61005, Korea
[3] Materials Center for Energy Department, Surface Technology Division, Korea Institute of Materials Science (KIMS), 797 Changwondaero, Sungsan-gu, Changwon, Gyeongnam 51508, Korea
* Correspondence: bjcho@chungbuk.ac.kr; Tel.: +82-(0)43-261-2417
† These authors contributed equally to this work.

Received: 11 July 2019; Accepted: 8 August 2019; Published: date

1. Fabrication process and band diagram for Fermi-level pinning with various metals

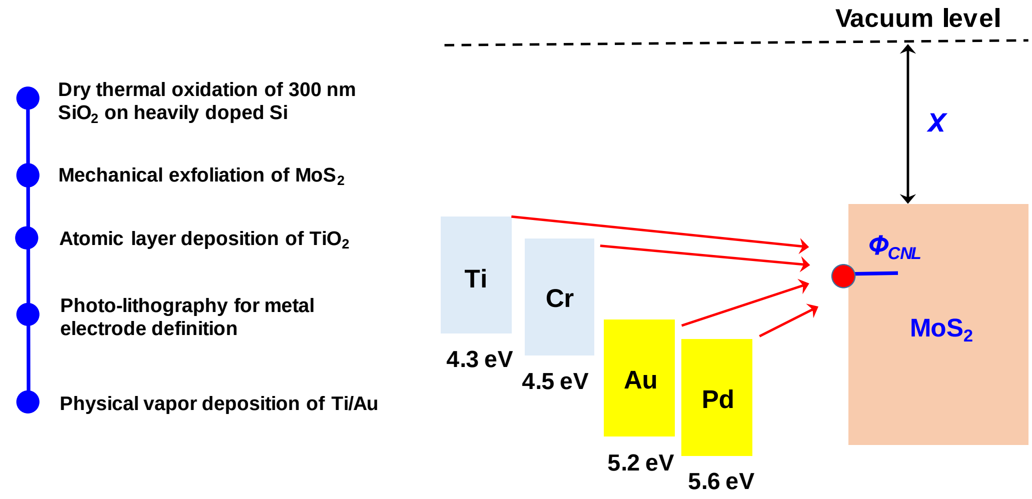

Figure S1. Fabrication process and band diagram for Fermi-level pinning with various metals.

Figure S1 shows fabrication process and the schematic for Fermi-level pinning in MoS_2. This is corresponding to the previous study [1].

2. Transfer and output curves for MoS_2 FETs without and with a TiO_2 layer

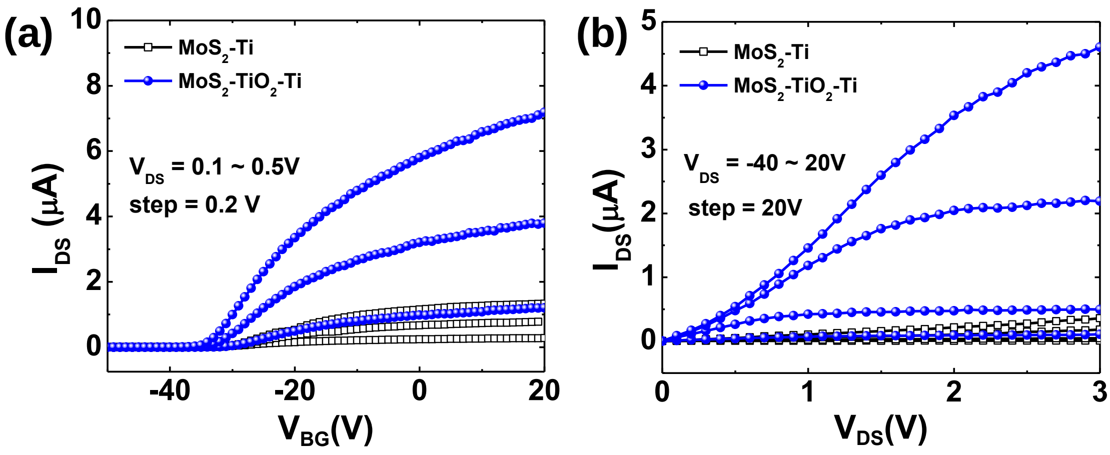

Figure S2. Transfer and output curves for MoS_2 FETs without and with a TiO_2 layer.

Figure S2 shows transfer and output curves for MoS_2 FETs without and with a TiO_2 layer. In the transfer curve, V_{BG} sweep was -50 ~ 20 V with V_{DS} = 0.1 ~ 0.5 V with 0.2 V of step. Improved performance was shown in the device with a TiO_2 layer, showing increased drain current. In the output curve, increased drain current and reduced series resistance (R_{SD}) were observed in the device with a TiO_2 layer.

3. Field-effect mobility (μ_{FE}) for MoS_2 FETs without and with a TiO_2 layer

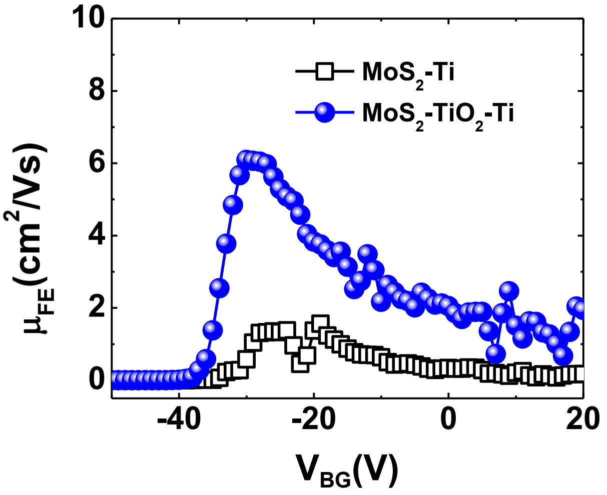

Figure S3. Field-effect mobility (μ_{FE}) for MoS_2 FETs without and with a TiO_2 layer.

Figure S3 shows field-effect mobility (μ_{FE}) for MoS_2 FETs without and with a TiO_2 layer. μ_{FE} values are changed as a function of V_{BG}, featuring maximum transconductance (g_m)

4. Transfer curves for WS_2-Ti, WS_2-TiO_2-Ti, WS_2-Pd, and WS_2-TiO_2-Pd structured FET device

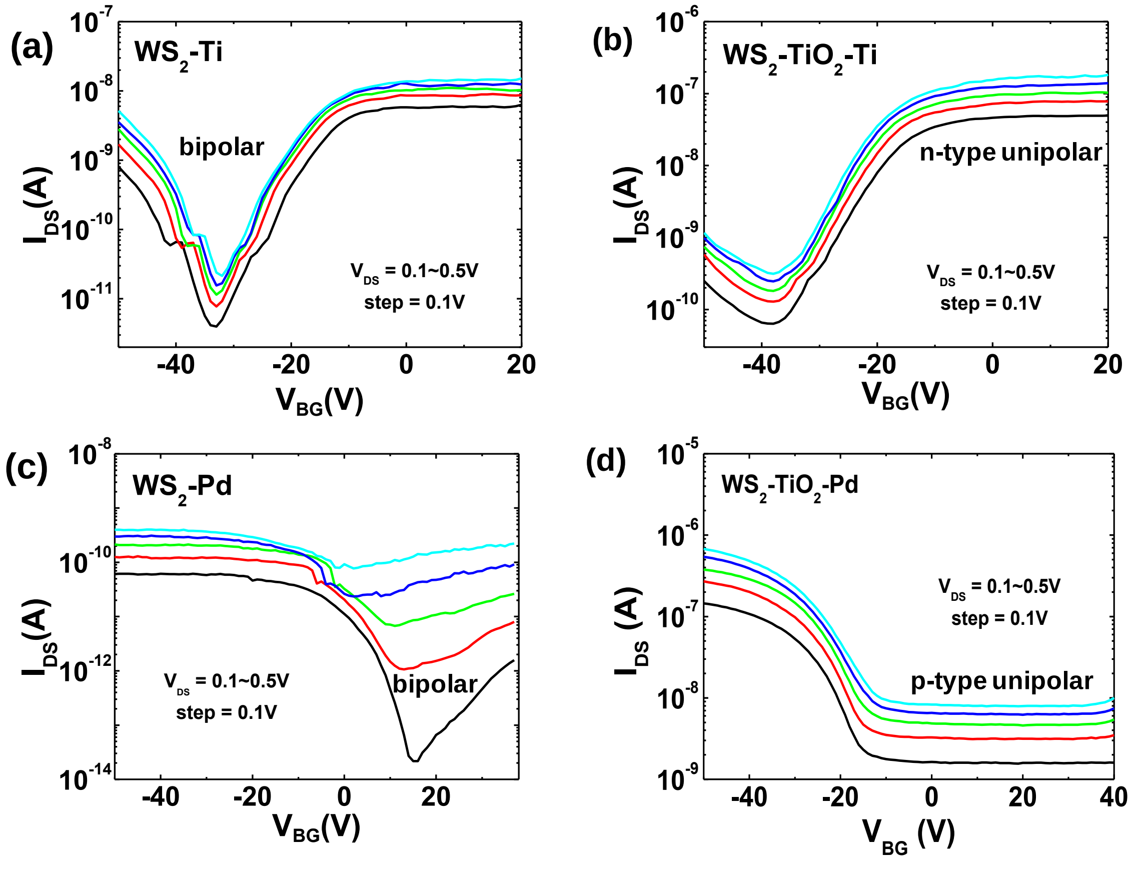

Figure S4. Transfer curves for WS_2-Ti, WS_2-TiO_2-Ti, WS_2-Pd, and WS_2-TiO_2-Pd structured FET devices.

Figure S4 shows the transfer curves for WS_2-Ti, WS_2-TiO_2-Ti, WS_2-Pd, and WS_2-TiO_2-Pd stack FETs. By inserting a TiO_2 layer, improved drive current was observed. Interestingly, type change behavior was observed from bi-polar to uni-polar behavior. Bi-polar behavior was observed from WS_2-Ti stack and it was changed to n-type unipolar behavior by using a TiO_2 layer. For Pd contact electrode, it was changed from bipolar to p-type unipolar behavior with the help of a TiO_2 layer. Previously, WS_2 FET have shown bipolar behavior and using a TiO_2 layer offers a benefit to achieve unipolar behavior which is usually used for an integrated circuit [2].

5. Energy band diagrams for TMDC-Ti and TMDC-TiO_2-Ti structures

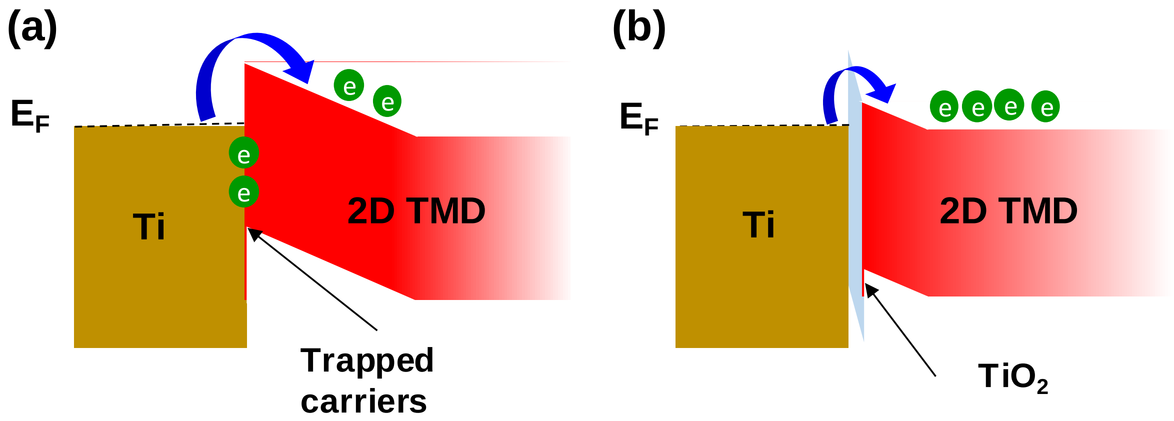

Figure S5. Energy band diagrams for TMDC-Ti and TMDC-TiO_2-Ti stacks.

Figure S5 shows band diagram for TMD-Ti and TMD-TiO_2-Ti stacks. The role of a TiO_2 layer is a buffer layer to cover dangling bonds to induce both Fermi-level pinning and device performance deviation. The effective Schottky barrier height becomes decreased in the TMD-TiO_2-Ti structure, leading to the increase in the electron conduction.

6. Bias stress measurement sequence

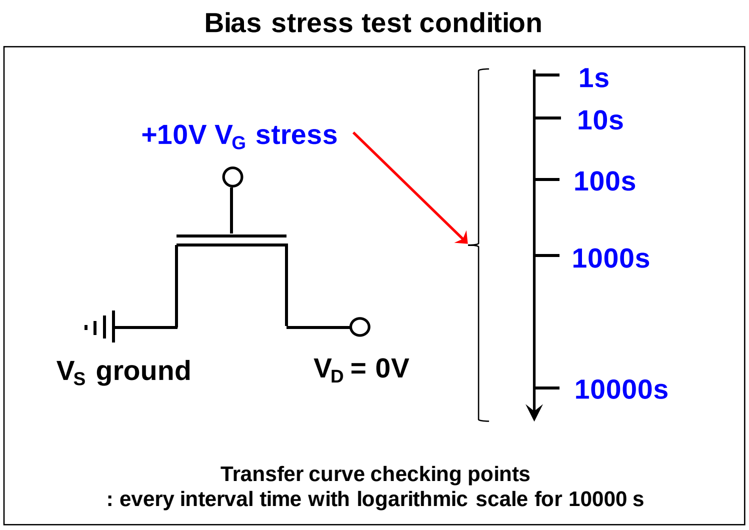

Figure S6. Bias stress measurement sequence.

Figure S6 shows the stress measurement set up environment and sequence. We followed the conventional repetitive stress-measurement-stress sequence. The stress measurements were conducted for 10000s and transfer curve was achieved every interval time with logarithmic scale (1s, 10s, 100s, 1000s, 10000s).

References

[1] C. Kim, I. Moon, D. Lee, M. S. Choi, F. Ahmed, S. Nam, Y. Cho, H.-J. Shin, S. Park, and W. J. Yoo, Fermi Level Pinning at Electrical Metal Contacts of Monolayer Molybdenum Dichalcogenides. *ACS Nano.* **2017**, *11*, 1588.

[2] W. S. Hwang, M. Remskar, R. Yan, V. Protasenko, K. Tahy, S. D. Chao, P. Zhao, A. Konar, H. Xing, A. Seabaugh and D. Jena, Transistors with chemically synthesized layered semiconductor WS2 exhibiting 105 room temperature modulation and ambipolar behavior. *Appl. Phys. Lett.* **2012**, *101*, 013107.
